# Blood-glucose levels in newborn piglets and the associations between blood-glucose levels, intrauterine growth restriction and pre-weaning mortality

**DOI:** 10.1186/s40813-019-0129-6

**Published:** 2019-10-01

**Authors:** Trude Staarvik, Tore Framstad, Mina Heggelund, Sunniva Brynjulvsrud Fremgaarden, Camilla Kielland

**Affiliations:** Faculty of Veterinary Medicine, Department of Production Animal Sciences, The Norwegian University of Life Sciences, Oslo, Norway

**Keywords:** Swine, Blood-glucose levels, IUGR, Mortality, Piglets

## Abstract

**Objectives:**

The aim of this study was to investigate the associations between blood-glucose levels in one-day-old-piglets (ODOP), intrauterine growth restriction (IUGR) and pre-weaning mortality in a commercial piglet-producing herd in Norway.

**Material and methods:**

The study was carried out in a non-crate commercial piglet-producing herd in Norway and 426 live born piglets from 31 litters were included. Piglets were blood-sampled, ear tagged, weighed and measured within 24 h after birth. Litter size, cross fostering and deaths until weaning were recorded. Blood was collected by vein puncture of *Vena subcutanea abdominis* and blood-glucose levels were measured using a handheld glucometer. Piglets were given an IUGR-score (1–3) based on head-morphology where a score of 3 is defined as an intrauterine growth restricted piglet.

**Results:**

Of the 426 live born piglets, 391 piglets survived until weaning, resulting in 8.22% pre-weaning mortality. Mean piglet weight in ODOP was 1.59 kg (SD = 0.36), and mean blood-glucose level was 5.48 mmol/l (SD = 1.44). IUGR score 3 piglets had lower blood-glucose levels (Coef. = − 1.7 mmol/l, *P* < .001) than normal piglets (IUGR score 1). Males had significantly higher blood glucose levels (Coef. = 0.23 mmol/l, *P* = .044) compared to females. There was a trend that blood-glucose levels in individual piglets were lower in large litters with − 0.07 mmol/l per extra piglet born (*P* = .054). Piglets with blood-glucose levels in the second quartile had reduced risk of pre-weaning mortality (OR = 0.32, *P* = .046) compared to piglets with blood-glucose levels in the lower quartile. This is also true for piglets in the third and fourth quartile (OR = 0.13, *P* = 0.004).

**Conclusion:**

This study identified IUGR to be associated with low blood-glucose levels in ODOP. It also found increased pre-weaning mortality in ODOP with low blood-glucose. By identifying IUGR piglets by the shape of their head, piglet producers may reduce pre-weaning mortality by making sure these piglets get enough colostrum, milk or supplement feeding (i.e. energy).

## Introduction

The intensification of husbandry leads to more effective animal production. This has resulted in hyper-prolific sows with large litter sizes and an increase in piglets weaned per sow. Even though there has been a continuing aim to reduce the pre-weaning mortality, piglet mortality is still a huge, multifactorial welfare problem all over Europe [1]. Reducing piglet mortality will increase the welfare of the piglets and be of economic benefit for the farmer. The two main causes of piglet mortality are trauma and starvation [2–4]. According to a review by Muns et al. most piglets die during the first 72 h after birth [2], hence resulting in the most critical period in a piglet’s life.

Piglets are born with limited body fat and without brown adipose tissue [5]. Body fat and brown adipose tissue are both vital in regulating body temperature through non-shivering thermogenesis [6]. Therefore, piglets have to produce heat by shivering, which requires energy from blood-glucose. In addition, piglets are born with an immature gluconeogenesis [7]. Therefore, they may not be physiologically capable of producing enough glucose to maintain normoglycemia in the first hours after birth. In this period, they are relying on glycogen storages and intake of colostrum or milk to maintain normoglycemia. Colostrum is therefore vital as an energy-supply, in addition to being important for immunity. If piglets do not feed, the blood-glucose levels will decrease to hypoglycemic levels (blood glucose < 2.2 mmol/l) within 24–36 h [7]. According to Theil et al., piglets are born with glycogen storages sufficient for just 16 h post-partum [8]. Hypoglycemic piglets will become lethargic, weak and in severe cases, it will result in coma or death [7]. Weak and lethargic piglets have a higher risk of being crushed by the sow when she moves around in the pen, in addition to the risk of dying from hypoglycemia itself. This raises questions on the association between blood-glucose levels in young piglets and pre-weaning mortality.

The positive relationship between colostrum intake and blood-glucose levels has previously been identified [9]. One study found birth weight to be an important factor influencing individual colostrum intake [10]. However, another study found only 5.9% of blood-glucose levels in one day old piglets to be explained by weight gain since birth [11]. This indicates that there are factors other than just colostrum intake and weight gain that influence blood-glucose levels in piglets.

High birth weight is associated with higher survival rate [12]. However, increased litter size is associated with a decrease in individual piglet birth weight [12]. Piglets with low birth weight and low body mass index (BMI) have a large body surface, and this makes them especially prone to hypothermia [13]. In addition to low birth weights, increased litter size also causes reduced uterine blood flow per fetus [14], which can result in intrauterine growth restriction [15]. Further on, IUGR is associated with reduced piglet survival [16]. IUGR piglets are observed to ingest reduced amounts of colostrum [17] which may have an effect on blood-glucose levels.

We hypothesize that low-blood-glucose levels are associated with piglet pre-weaning mortality. We also hypothesize that there are factors other than just colostrum intake that influence blood-glucose levels in one-day-old piglets (ODOP). Such factors might be litter size, BMI or IUGR.

The aim of this study was to investigate the association between blood-glucose levels in ODOP, IUGR and pre-weaning mortality in a commercial piglet-producing herd in Norway.

## Materials and methods

Permission to conduct this study was obtained from the Norwegian Food Safety Authorities in accordance with The Norwegian Regulation on Animal Experimentation §6.

### Study design and study population

The study was carried out in a commercial piglet-producing herd in Eastern Norway in January 2017. The herd selection was based on location and owner-compliance. The herd consists of 110 sows, split into two batches, with farrowing every 11th week.

In the study period, 55 sows farrowed. A litter was included in the study if it was born at a time to be approximately one-day-old when the research team was present at the farm. The researchers were present during daytime every day for the 7 days when sows were expected to farrow. This resulted in samplings from 426 live-born piglets from 31 litters that were included in the study. The rest of the sows farrowed at a time when researchers were not present to sample data from the litter.

The piglets were born from Norwegian Landrace X Yorkshire Topigs z line (ZL) or Norwegian Landrace X Yorkshire (YL) sows, by Duroc boars. Recently, the Norwegian piglet producing herds changed from Yorkshire to Yorkshire Topigs z-line genetics; hence, there was a mixture of sow genetics available in the study herd. The parity of the sows ranged from 1st to 7th parity. Of the sows, 12 were 1st or 2nd parity, 9 were 3rd parity and 10 were 4th parity or older.

### Housing

**Fig. 1:**
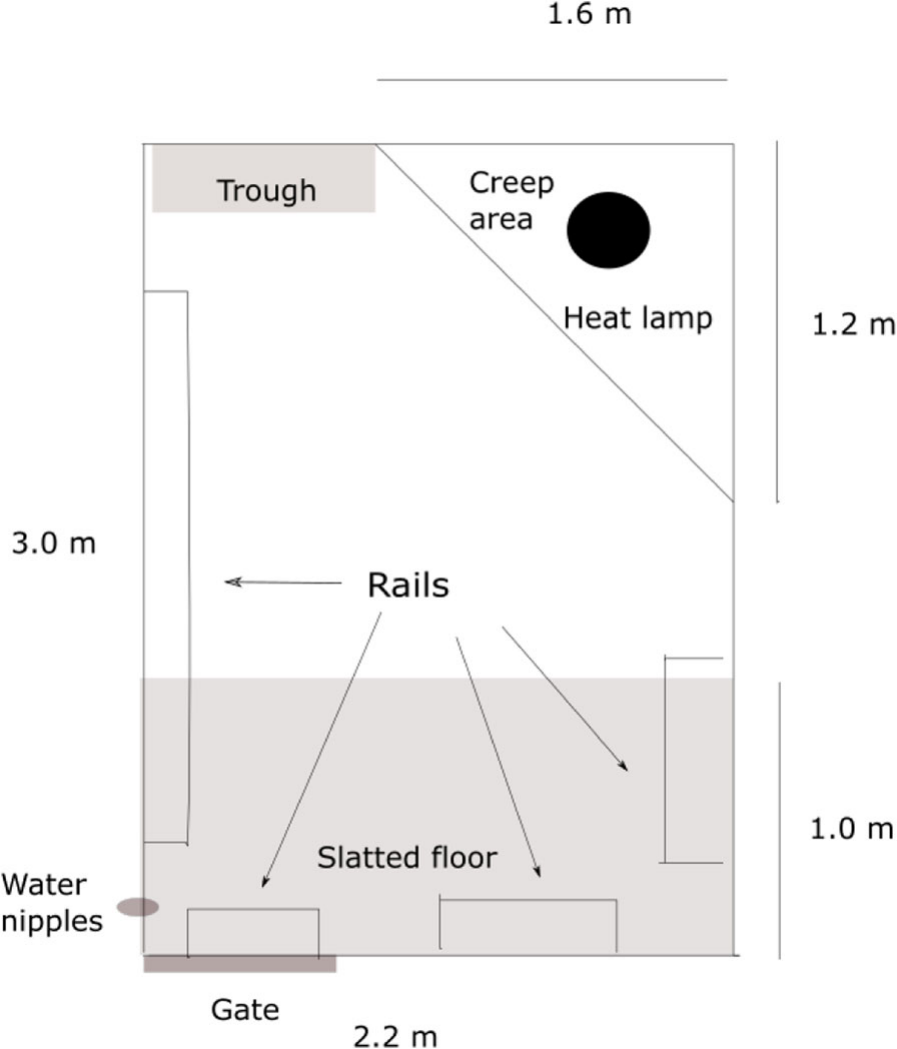
Design of the farrowing pen. Illustration of the farrowing pen used in this study. The creep area had heated floors, in addition to a heat lamp. Water nipples were distributed in two heights, one for the sow and one for piglets. Sows were loose housed during the entire production cycle

During gestation, the sows were loose-housed in groups, with deep straw bedding. These pens had self-closing feeding stalls. A few days before expected farrowing, the sows were moved from the gestation unit into two identical farrowing units. The units had 4 rows of pens, each row consisting of 7 pens. The farrowing pens were non-crated pens 2.2 × 3.0 m (Fig. 1). These pens had a triangular creep area (1.6 × 1.2 m) inaccessible for the sow. The creep area had heated floors and a heating lamp. Farrowing rails were present in the pen. Inside the creep area, sawdust was used as bedding material. The temperature of the heated floor in the creep area was regulated on individual rows of pens. Mean temperature in the creep area was 27.1 (SD = 1.8) degrees Celsius. Straw was provided as nest building material in the farrowing pen one day before expected farrowing. Sows and piglets had ad libitum access to water through water-nipples. In the farrowing pens, one water nipple was at a height of approximately 10 cm, and one approximately 90 cm.

### Sow management

The sows were fed commercial feed mixed with water. During gestation, the sows were fed a gestation diet (8.72 MJ NE/kg feed, 6.2 g total lysine/kg feed) twice daily. From gestation day 80, the amount of feed was gradually increased to 3.3 kg daily. After farrowing, the sows were fed a lactation diet (9.97 MJ NE/kg feed, 8.8 g total lysine/kg feed). The sows were fed twice daily the first 3 days after farrowing, then three times daily the rest of the lactation period.

Before insemination, all sows were vaccinated against Parvovirus and *Erysipelothrix rhusiopathiae,* and during gestation, they received a vaccine against *Escherichia coli*.

Farrowing was allowed to start naturally, and human intervention and the use of oxytocin were kept to a minimum. Colostrum intake in piglets was not recorded.

### Piglet management

The day after birth, all piglets had their teeth grinded by the producer, at a different time to that of the data sampling. Teeth grinding is permitted up to 7 days of age according to Norwegian legislation [18]. At three or four days of age the piglets were given an intramuscular iron-injection; 1 ml Gleptoferron Fe^3+^ 200 mg/ml (Gleptosil vet.® Ceva Santé Animale). Cross fostering was performed when piglets born into big litters were born at approximately the same time as another sow with a smaller litter. When cross fostering was performed, piglets were fostered into smaller litters with similar sized littermates, and these piglets were categorized as cross-fostered. Cross-fostering was aimed to occur within the first two days after birth. If a piglet was cross-fostered after data sampling, there is no record of when cross fostering occurred. Both birth mother and foster mother were registered for cross-fostered piglets. Before one week of age, all male piglets were surgically castrated under local anesthesia and pain relief (a non-steroidal anti-inflammatory drug (NSADIs)) by a veterinarian in accordance with Norwegian legislation. From the second week after birth, the piglets were offered peat added iron and piglet feed (10 MJ NE/kg feed, 14.1 g total lysine/kg feed) in the creep area.

Some of the sick piglets were fostered together in pens with a fostering sow (*N* = 21 piglets). In addition to milk from the sow, piglets in these pens were fed a commercial milk substitute. These sick piglets were also included in the study. The majority of these piglets had infectious arthritis, and they were treated with intramuscular injections of benzylpenicillin.

### Data collection

Environmental variables recorded were temperature (°C) in the pens and creep areas.

Sow and litter variables recorded were parity of the sow, if manual assistance or oxytocin were used during farrowing, and litter size including the number of live born and stillborn piglets. Body condition scoring of the sows was performed two days after farrowing, using a Norwegian standard body condition scoring protocol [19]. Mean sow body condition score two days after farrowing was 3.29 (SD = 0.35) on the scale ranging from 1 to 5.

ODOP had their blood drawn, and were then sexed, weighed and given an IUGR-score. Crown to rump length was measured and they were ear tagged with individual numbers. Mean age at data collection was 19.45 (SD = 7.86) hours. Blood sampling was performed first to minimize the impact of stress on blood-glucose levels. A droplet of blood was collected by puncture of Vena subcutanea abdominis [20] and blood-glucose levels (mmol/l) were analyzed with the handheld glucometer Contour next USB (Bayer Consumer Care AG, Peter Merian-Strasse 84, 4052 Basel, Switzerland). If it was not possible to get enough blood form Vena subcutanea abdominis, blood was collected by puncture of Vena cephalica. Weights were recorded in grams rounded to the nearest 10 g (EKS premium 8006 GR-ST), and length measured in centimeters from Crista nuchalis to the first coccygeal vertebrae (crown to rump length). The piglets were given an IUGR-score based on head morphology as described by Chevaux et al. and Hales et al [16, 21]; the criteria that characterize growth restriction in piglets are 1) steep, dolphin-like forehead, 2) bulging eyes, and 3) wrinkles perpendicular to the mouth. Piglets with no sign of intrauterine-growth restriction were given score 1 (normal), piglets with one or two characteristics of intrauterine growth restriction were given score 2 and piglets with all the characteristics of intrauterine growth restriction were given score 3. Piglets with IUGR score 3 are hereafter called IUGR-piglets.

Piglets were weighed again at three weeks of age and at weaning at 34.4 (SD = 1.24) days of age.

During the lactation period, the farmer recorded the individual ID-number and date of death of deceased piglets.

### Statistical analyses

All statistical analyses were performed using STATA (Statistical software (StataCorp. 2015. *Stata Statistical Software: Release 14*. College Station, TX: StataCorp LP).

For the descriptive analyzes scatter plots and histograms were used.

Statistic significant results were defined as *p* < .05, and *p* < .10 was defined as a trend.

### Model building

Initially, the continuous variables were checked for normality and the associations between outcome/dependent variables and explanatory variables were evaluated visually using graphs.

To test our hypotheses, two statistical models were used. The first model (Blood-glucose model) was a multilevel mixed effect linear regression model with blood glucose levels (mmol/l) at day one as the outcome. The second model (Mortality-model) was a multilevel mixed effect logistic regression model with piglet dead or alive at weaning (binominal) as the outcome. Individual piglets were nested to sow, and sow was therefore included as a random effect in both models.

Univariate regression analysis was used to determine which explanatory variables to include in the final models. Explanatory variables tested in both models were a mixture of piglet and sow variables. Piglet variables tested were weight, length, sex, BMI (kg/m^2^), cross-fostered (Y/N) and IUGR score [1–3]. In the mortality-model, blood-glucose in ODOP was also included as an explanatory variable. Sow variables included were litter size and parity of the sow. Variables with *p* < .2 were considered for the final model. Variables were included in the model if they were statistically significant (*p* < .05), or if they were considered confounders.

In the blood-glucose model, explanatory variables included were IUGR score, BMI, gender and litter size. In the Mortality-model, explanatory variables included were blood-glucose level in ODOP categorized into quartiles, cross-fostered (Y/N), litter size and BMI. Litter size included stillborn piglets in both regression models. BMI was included instead of weight and length since they were highly correlated (0.80, *p*<. 001), in addition, by including BMI, the model improved. Possible improvement of the model was assessed by using a likelihood ratio test. Evaluation of the model assumptions for the random effects was by visual inspection of normal-probability plots of the standardized residuals.

#### Missing values

Four piglets were not present in the herd at weaning, nor recorded dead by the farmer, hence they were assumed deceased without being recorded.

## Results

Of the 426 live born piglets, 391 were alive at weaning, resulting in 8.22% pre-weaning mortality. Mean litter size including stillborn was 16.1 piglets (SD = 3.7). Mean number of stillborn piglets in each litter was 1.05 (SD = 1.5). Mean weight in ODOP was 1.59 kg (SD = 0.36) ranging from 0.56 kg to 2.83 kg. Mean blood glucose level in ODOP was 5.48 mmol/l (SD = 1.44), ranging from 1.2 mmol/l to 10.6 mmol/l. Of the 426 piglets, 48% (*N* = 203 piglets) were given IUGR-score 2, and 6.3% (*N* = 27 piglets) were given IUGR-score 3. Of piglets born into litters with more than 15 piglets (*N* = 257 piglets) 8.95% were IUGR-piglets, whereas only 2.4% born into a litter with 15 piglets or less (*N* = 169 piglets) were IUGR piglets. 35.9% of live-born piglets (*N* = 153 piglets) were cross-fostered and mean litter size after cross fostering was 14.5 piglets (SD = 1.6). Of these cross-fostered piglets, 21 (4.9%) were placed in hospital pens. Only 18 of the 153 cross-fostered piglets were cross-fostered before data collection at day 1. In total, 5 sows received assistance during farrowing (16.1% of sows). Mean blood glucose levels in these litters were 5.2 mmol/l (SD = 1.6). Of these, 2 received both manual assistance and an injection of oxytocin, whereas the rest only received manual assistance. A summary of descriptive statistics is presented in Table 1.

**Table 1:** Descriptive statistics of piglets in a commercial piglet-producing herd in Norway

Variable	*n*	*Median*	*Min*	*Max*	*Mean*	*SD*
Litter size	31	16.0	7.0	23.0	16.1	3.7
Weight (kg)	426	1.6	0.6	2.8	1.6	0.4
Length (cm)	426	25.0	17.5	30.0	24.8	2.0
BMI	426	25.4	13.1	51.9	25.5	3.8
Blood-glucose levels (mmol/l) in one-day-old piglets	426	5.5	1.2	10.6	5.5	1.4
Weight 3 weeks (kg)	391	6.3	1.5	10.6	6.5	1.7
Weight weaning (kg)	391	10.8	2.8	17.0	10.8	2.7

**Fig. 2:**
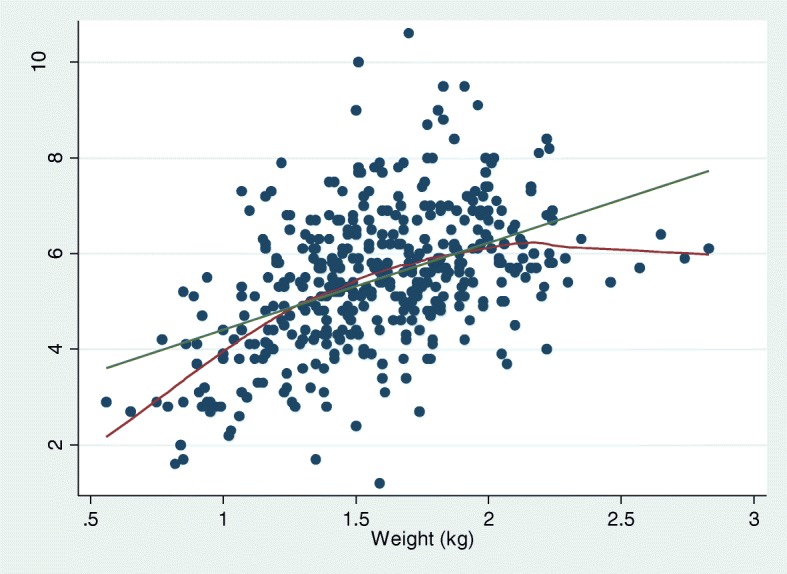
Correlation between weight and blood-glucose levels in one-day-old piglets (ODOP), Blood was collected from *Vena subcutanea abdominis* from one-day-old piglets (*N* = 426) and analyzed with a handheld glucometer. Piglets were weighed; heavier piglets were shown to have higher blood-glucose levels. The green line is the fitted values, which shows the best-fit straight line. This line shows a clear correlation. However, the red line is the lowess smoothing line, and it is the line that fits the data best. This line demonstrates that if the piglet weighs more than 2 kg there is no additional gain on blood-glucose levels. Piglets less than 1 kg are at risk of hypoglycemia

**Fig. 3:**
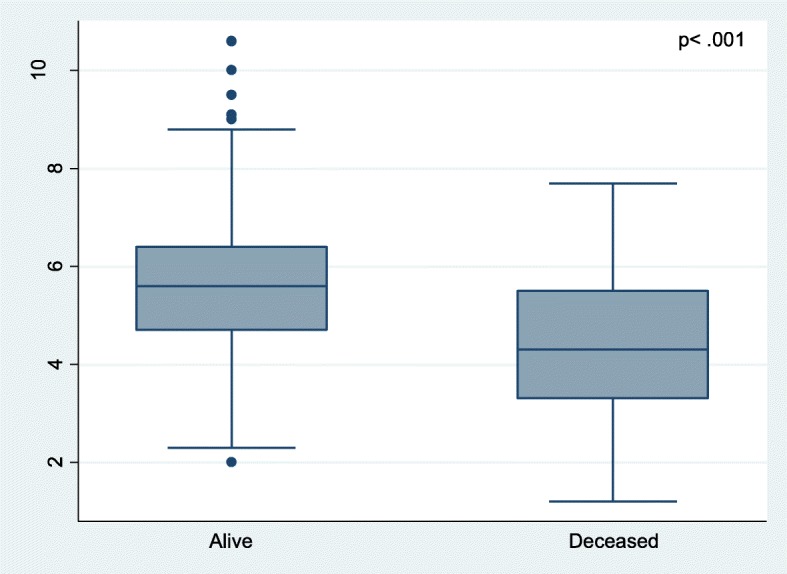
Comparison of blood-glucose levels at day one in piglets alive and deceased at weaning. Blood was collected from *Vena subcutanea abdominis* from one-day-old piglets (*N* = 426) and analyzed with a handheld glucometer. Piglets who did not survive until weaning had significantly lower blood-glucose levels the day after birth compared to piglets who survived until weaning

The association between blood-glucose levels and weight in ODOP is presented in Fig. 2, with a fitted line and a lowess smoothing line (R^2^ = .21). The association between blood-glucose levels in ODOP and mortality until weaning is presented in Fig. 3. Mean blood-glucose level in the group of surviving piglets was 5.57 mmol/l, and mean blood-glucose levels in the group of deceased piglets was 4.37 mmol/l, hence piglets who died before weaning had 1.2 mmol/l (*p* < .001.) lower blood-glucose level at day one compared to piglets who survived until weaning.

**Fig. 4:**
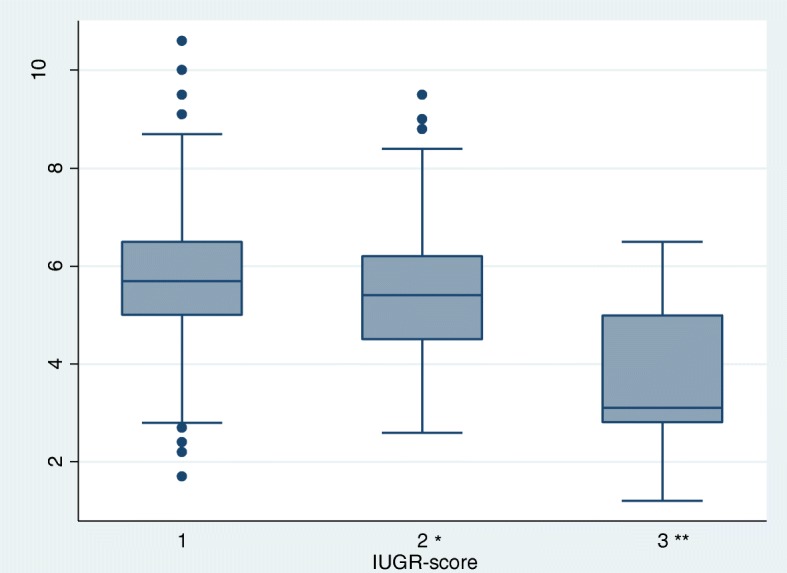
Blood-glucose levels in one-day-old piglets (ODOP) with different intrauterine growth restriction (IUGR) score. Blood was collected from *Vena subcutanea abdominis* from one-day-old piglets (*N* = 426) and analyzed with a handheld glucometer. Piglets were given a score [1–3] for intrauterine growth restriction (IUGR) based on the shape of their head. IUGR-piglets had significantly lower blood-glucose levels the day after birth compared to normal piglets. 196 piglets were given IUGR score 1, 203 piglets were given IUGR-score 2, and 27 piglets were given IUGR-score 3. * *p* = 0.038, ** = *p* < 0.001

IUGR piglets had reduced blood-glucose levels compared to normal piglets (Fig. 4).ODOP with IUGR-score 3 had 1.7 mmol/l lower blood-glucose levels compared to normal piglets (*p* < .001).

### Blood-glucose model

Results from the linear regression addressing blood-glucose levels in ODOP are presented in Table 2. Interestingly, when controlling for litter size, BMI and gender, an IUGR piglet (score 3) had 1.7 mmol/l lower blood-glucose levels compared to a non-IUGR piglet (*p* < .001). This lower blood-glucose level was also the case for piglets with IUGR score 2 (Coef. = − 0.25, *p* = .038). IUGR-score 2 and 3 and big litters were associated with reduced blood-glucose levels in ODOP in the investigated commercial piglet producing herd. Males had significantly higher blood glucose levels (Coef. = 0.23 mmol/l, *p* = .044) compared to females. It was a trend that bigger litters were associated with lower blood glucose levels in individual piglets as each extra piglet born were associated with a 0.07 mmol/l lower blood glucose level (*p* = .054). BMI was not significantly associated with blood-glucose levels but was included in the model.

**Table 2:** Linear regression with blood-glucose levels (mmol/l) in one-day-old piglets as the outcome

Variable	Estimate	SE^1^	*p*-value	95% CI^2^
Intercept	6.18	0.74	< 0.001	[4.74, 7.64]
Intrauterine growth restriction score (IUGR-score)				
IUGR score 1 (normal)	Baseline			
IUGR score 2	−0.25	0.12	0.038	[−0.49, −0.01]
IUGR score 3	−1.67	0.25	< 0.001	[−2.17, −1.18]
Gender				
Female	Baseline			
Male	0.23	0.11	0.044	[0.01, 0.45]
BMI	0.02	0.01	0.219	[−0.01, 0.05]
Litter size	−0.07	0.03	0.054	[4.74, 7.64]

*n* = 426 piglets from 31 litters

1– standard error, 2 – confidence interval

Presented with coefficients, standard error (SE) and *p*-values and 95% confidence interval.

### Mortality-model

Detailed results from logistic regression mortality-model are presented in Table 3. Piglets with blood-glucose levels in the second quartile had reduced risk of dying (OR = 0.32, *p* = .046) compared to piglets with blood-glucose levels in the lower quartile. This is also true for piglets in the third and fourth quartile (OR = 0.13, *p* = .004). Cross-fostering was associated with reduced risk of pre-weaning mortality compared to the piglets that were not cross-fostered (OR = 0.11, *p* < .001). Litter size (*p* = .760) and BMI (*p* = .842) were not significantly associated with piglet mortality, but they were included in the final model because they are believed to be confounders, and including them improved the model.

**Table 3:** Logistic regression with piglet deceased or alive at weaning as outcome

Variable	Odds ratio	SE^1^	*p*-value	95% CI^2^
Intercept	7.17	17.98	0.433	[0.05, 981.44]
Blood-glucose level in one-day-old piglets (mmol/l)				
Lower quartile	Baseline			
Second quartile	0.32	0.18	0.046	[0.11, 0.98]
Third quartile	0.13	0.09	0.004	[0.03, 0.52]
Upper quartile	0.13	0.09	0.004	[0.03, 0.52]
Cross-fostered				
No	Baseline			
Yes	0.11	0.07	0.001	[0.03, 0.38]
Litter size	0.97	0.11	0.76	[0.78, 1.20]
BMI	0.99	0.05	0.842	[0.89, 1.10]

*n* = 426 piglets from 31 litters

1 - Standard error, 2 – Confidence interval,

Presented with odds ratios, standard error (SE), *p*-values and 95% confidence intervals (CI).

## Discussions

This study confirms that low blood-glucose in ODOP increases the risk of mortality before weaning, even when controlling for BMI, litter size and cross-fostering. This is in accordance with other studies who found decreased blood-glucose levels at day 1 after birth in piglets who died before weaning [9, 22]. In addition, this study identified IUGR piglets and big litters as risk factors for low blood-glucose in ODOP.

This study was carried out in one commercial piglet-producing herd, and with conveniently chosen litters. Optimally the study would include multiple herds and randomly chosen litters to eliminate management as a potentially confounding factor. Still, since the study was carried out in a commercial herd, and the researchers did not alter management, one can assume the results are representative for similar commercial piglet-producing herds in Norway.

The method used to analyze blood-glucose is minimally stressful for the piglets, efficient and minimally invasive. For human use, the glucometer used is proven to be highly accurate [23]. One study in neonatal foals found a similar glucometer to be accurate in estimating blood-glucose levels compared to a standard laboratory method [24]. Nunnelley et al found that glucometers could reliably estimate the glucose levels in fluorinated plasma in pigs [25]. This gives reason to believe the method used is accurate estimating blood-glucose levels in newborn piglets.

Undernutrition of the mother causes reduced birth-weights in the offspring [26]. However in pigs, given sows are not undernourished, Theil et al concluded that maternal nutrition does not affect fetal glycogen, as no studies have managed to alter fetal glycogen through altering maternal diets [27]. The sows in this study were fed increasing amounts of high-quality commercial feed from day 80 of gestation, facilitating fetal growth. Before day 80, they were fed maintenance. This makes it unlikely that sow nutrition affected the blood-glucose of ODOP.

Pre-weaning mortality in this study was 8.22%, which is below the Norwegian average of 12.4% pre-weaning mortality [28]. The below-average mortality is likely a result of management in the herd rather than an effect of close monitoring during the neonatal period or interference by the research group. A possible contributor to this below-average mortality could be the sick piglets fostered in separate pens. One can question if these piglets would have survived if they had remained in their original litter without the extra care.

The observed temperature in the creep area was below the recommended 30 degrees Celsius. One possible explanation for this is the outside temperature. Mean outside temperature in this area in January 2017 was minus 2.5 degrees Celsius. A cold environment makes the piglets prone to hypothermia. Piglets are born without brown adipose tissue, and therefore have to produce heat through shivering [5]. This process requires energy from blood-glucose, and may be one of the reasons for the observed increased mortality associated with low blood-glucose levels in ODOP. Cold piglets are also more likely to lie near the mother to maintain normal body temperature, and this makes them prone to crushing by the sow.

### Intrauterine growth restriction

Placental insufficiency is the leading cause of intrauterine growth restriction in animals [29]. However, Hendrix and Beghella describe a list of non-placental causes of IUGR in humans [30]. Fetal causes may include genetic diseases, infections, malformations or cord abnormalities. Maternal causes of IUGR may include disease, toxicity or malnutrition. There were no observed cases of disease or malnutrition in this study. IUGR alters the physiology of multiple organ-systems, but only those relevant for this study will be discussed further.

### Blood-glucose levels in IUGR piglets

Intrauterine growth restriction was a risk factor for low blood-glucose in ODOP in this study. This is in accordance with another recent study [17]. Amdi et al. found no difference in blood-glucose levels between IUGR piglets and normal piglets on whole blood but found plasma-glucose levels to be lower in IUGR piglets [31].

Multiple authors have studied the intestines of IUGR piglets, Wang et al described a longer and thinner small intestine with reduced absorption area [32]. D’Inca described a delay in the normal gut development in IUGR piglets compared to normal piglets [33]. Combined with the findings of Amdi et al [17] who describes an insufficient colostrum intake in IUGR piglets, it is likely that IUGR piglets have an insufficient absorption of glucose from the intestine, which will result in lower blood-glucose levels. Also, Chen et al found important proteins in metabolism to be lower in the placenta of IUGR piglets compared to the placenta of normal piglets [34], which likely will result in IUGR-piglets being born with reduced glycogen storages. Wang et al described that IUGR piglets have different expression of proteins important for energy metabolism [35], hence it is reasonable to believe that IUGR piglets have an impaired ability to produce glucose from glycogen. The combination of reduced glycogen storages and an impaired ability to produce glucose from glycogen may explain the reduced blood-glucose levels in IUGR piglets found in this study.

### Litter size

The results of this study indicated that litter size is associated with the blood-glucose level of the ODOP though not statistically significant. Increased litter size may increase the within-litter competition both pre- and postpartum. Additionally, uterine blood flow per fetus decreases when litter size increases [14]. Reduced blood flow to each fetus may result in less nutrients available for each piglet in utero, and may cause the piglets to be born with reduced storages of glycogen compared to piglets in smaller litters. Reduced blood flow to each fetus is likely to affect piglet growth in the uterus, and thus consequently affect intrauterine growth restriction. This is in accordance with our study where 8.95% of piglets born into litters larger than 15 piglets were IUGR-piglet, and only 2.37% of piglets born into litters with 15 piglets or less were IUGR-piglets. Colostrum yield is not affected by litter size [36], and consequently less colostrum is available per piglet in large litters, which may result in reduced energy intake in individual piglets,

### Cross fostering

In this study, being cross-fostered was associated with reduced pre-weaning mortality. It is not possible to tell if these piglets would have survived if they had remained in their original litter. Being cross-fostered may have been favorable because piglets were fostered into smaller litters with similar sized littermates. The favorable effect of the cross fostering practice found in this study is supported by one study who found mortality of low-birth weight piglets to be higher in large litters with high-birthweight litter-mates [37], but another recent study found an increased risk of mortality in cross fostered piglets [38]. In this study, the fostering of sick piglets into hospital pens where they were given extra care probably resulted in reduced mortality compared to if they were not fostered in separate pens.

### Future work

The causes of piglet mortality were not investigated in this study, and future work needs to be done to investigate the association between blood glucose levels and causes of death. It would also be interesting to investigate if similar results are found when also controlling for colostrum intake in individual piglets.

## Conclusion

To conclude, intrauterine growth restriction was a risk factor for low blood-glucose in ODOP in this study and low blood-glucose levels in ODOP are significantly associated with pre-weaning mortality. The farmer can easily recognize IUGR-piglets by their characteristic head-shape. By identifying these high-risk piglets, the farmer can take action to prevent unnecessary mortality. In a commercial setting, the most convenient action is to make sure these piglets get adequate colostrum and milk, for example by letting these piglets suckle while the rest of the litter is in the creep area, to reduce the competition at the udder from littermates. Commercial supplements may also be considered. By providing extra care, the farmer can reduce pre-weaning mortality and improve piglet welfare and economic gain. In an experimental setting, it would be interesting to assess the effect of oral glucose supplements or an injection of glucose.

## Data Availability

Datasets from which the results in this study are based upon are available from the authors upon reasonable request.
